# Identification and Characterization of *Staphylococcus aureus* Strains with an Incomplete Hemolytic Phenotype

**DOI:** 10.3389/fcimb.2016.00146

**Published:** 2016-11-18

**Authors:** Haifang Zhang, Yi Zheng, Huasheng Gao, Ping Xu, Min Wang, Aiqing Li, Minhui Miao, Xiaofang Xie, Yimai Deng, Huiqin Zhou, Hong Du

**Affiliations:** ^1^Department of Clinical Laboratory, The Second Affiliated Hospital of Soochow UniversitySuzhou, China; ^2^Department of Clinical Laboratory, The Fifth People's Hospital of SuzhouSuzhou, China

**Keywords:** *Staphylococcus aureus*, incomplete hemolytic phenotype, hemolysin, drug resistance, virulence

## Abstract

*Staphylococcus aureus* is a common pathogen causing both hospital and community-acquired infections. Hemolysin is one of the important virulence factors for *S. aureus* and causes the typical β-hemolytic phenotype which is called complete hemolytic phenotype as well. Recently, *S. aureus* with an incomplete hemolytic phenotype (SIHP) was isolated from clinical samples. To study the microbiologic characteristics of SIHP, the special hemolytic phenotype of SIHP was verified on the sheep blood agar plates supplied by different manufacturers. Expression of hemolysin genes *hla, hlb, hlgC*, and *hld* of SIHP was detected by qRT-PCR and it was showed that expression of *hlb* in SIHP was obviously increased compared to the control *S. aureus* strains with complete hemolytic phenotype (SCHP), while the expression of *hla, hlgC*, and *hld* in SIHP was significantly decreased. In addition, the α-hemolysin encoded by gene *hla* was decreased obviously in SIHP compared to SCHP by western blot. All 60 SIHP strains were identified to be the methicillin resistant *S. aureus* (MRSA), and moreover these SIHP strains all contains *mecA* gene. The virulence gene *tst* were all present in SIHP, and the intracellular survival ability of SIHP was much greater than that of the gene *tst* negative *S. aureus*. We also found that IL-2, IL-6, and IL-17A secreted in the supernatant of SIHP infected macrophages increased significantly compared to *tst* negative control strains infected ones. MLST analysis showed that all of SIHP strains were classified into ST5 clone. To our knowledge, this study firstly showed that SIHP strains are a kind of methicillin resistant strains which express β-hemolysin highly and possess a potential high virulence, and it was suggested that SIHP should be paid more attention in hospital.

## Introduction

*Staphylococcus aureus* is an important human pathogen isolated from hospitalized patients worldwide, which causes both hospital and community-acquired infections (Lowy, 1998). This pathogen is the etiological agent of several different systemic infections, affecting skin and soft tissue, as well as musculoskeletal and circulatory systems (Lowy, 1998; Changchien et al., 2016). It was reported that *S. aureus* can survive in human monocyte-derived macrophages (Kubica et al., 2008). The virulence of *S. aureus* is closely associated with a variety of secreted enzymes and toxins produced by the bacteria (Otto, 2014). Hemolysin, leukocidin (Panton–Valentine leukocidin, PVL), and toxic shock syndrome toxin-1 (TSST-1), facilitating for damaging the red cell membrane, injuring phagocytic function of leukocytes, and inducing toxic shock syndrome respectively, are critical for the pathogenic processes of *S. aureus* (Löffler et al., 2010; Vandenesch et al., 2012; Berube and Bubeck Wardenburg, 2013; Andrey et al., 2015; Al Laham et al., 2015). Recent studies have demonstrated that hemolysin also participates in the formation of the *S. aureus* biofilm (den Reijer et al., 2016). The increasing prevalence of multidrug-resistant *S. aureus* strains within hospital and community environments further increases the dangers of *S. aureus* and poses a serious challenge for clinical therapy (Voss and Doebbeling, 1995; Evangelista Sde and de Oliveira, 2015).

Recently, a number of strains belonging to a class of *S. aureus* with an incomplete hemolytic phenotype (SIHP) have been found in our hospital. Hemolysis caused by these SIHP strains is significantly different from the complete hemolytic ring (β-hemolytic phenotype) produced in other *S. aureus* strains. However, these SIHP strains have not yet been identified and characterized comprehensively. To explore the microbiological characteristics of these SIHP strains, we collected 60 SIHP strains and studied them using multiple criteria including hemolytic phenotype, expression of the hemolysin gene, drug-resistance features, and virulence. This study demonstrates that SIHPs are methicillin resistant strains that highly express β-hemolysin and possess a high virulence potential.

## Materials and methods

### Bacterial strains

Sixty SIHP strains were isolated from patients admitted in the second affiliated hospital of Soochow University between 2013 and 2015. Duplicate samples from each patient were taken for analysis. These isolates were then cultured on Columbia sheep blood agar plates (CHROMagar Company, Shanghai, China) at 35°C in an atmosphere containing 5% CO_2_ (v/v). Strains identity as *S. aureus* were confirmed using the Phoenix-100 automated microbiology system (Becton, Dickinson and Company, USA). The control *S. aureus* strains, with complete hemolytic phenotype, were isolated from patients in our hospital during the same time period. Here, *S. aureus* with the complete hemolytic phenotype was called SCHP strains. The *S. aureus* ATCC25923 reference strain (Shanghai Center for Clinical Laboratory, China) has the complete hemolytic phenotype and acted as the control strain as well. The Medical Ethics Committee of Second Affiliated Hospital of Soochow University approved this study and all isolates were collected with patient consent in this study.

### Comparative analyses of incomplete hemolytic phenotype

The *S. aureus* strains were cultured on commercial blood agar plates from different companies (CHROMagar, Autobio Diagnostics Co., Ltd., China and BioMérieux, China) as well as self-prepared sheep blood agar plates using Columbia blood agar powder (OXIDE, UK). The bacteria were cultured at 35°C in an atmosphere containing 5% CO_2_ (v/v) for 24 h and then underwent serial passage. The hemolytic phenomenon was then observed. Clinically-isolated strains with complete hemolytic phenotype and the ATCC25923 reference strain were also observed as a control for comparative analyses.

### Determination of the mRNA levels of four hemolysin genes of *S. aureus* using reverse transcription real-time quantitative PCR (qPCR)

Total RNA extraction from the SIHP and the ATCC25923 reference strain was undertaken as previously described (Qin et al., 2014). Initially the cells were lysed using lysostaphin, lysozyme, and proteinase K. RNA was then extracted and purified using RNeasy Mini Kit (Qiagen) according to the manufacturer's recommended protocol. RNA quality and concentration was evaluated using the NanoDrop1000. RNA was then reverse-transcribed to cDNA using a reverse transcription kit (Thermo Fisher Scientific Inc., USA) according to the manufacturer's instructions. Briefly qPCR was conducted as follows: pre-denaturation at 94°C for 3 min, denaturation at 94°C for 30 s, annealing at 52°C for 30 s, elongation at 72°C for 40 s, a total of 45 cycles. Each sample had three technical and three biological repeats. The transcriptional profile of each of the four hemolysin genes and the *16s* rRNA gene was determined using the 2^−ΔΔ*Ct*^ method. The level of transcription was determined relative to the expression of the *16s* rRNA gene. The sequences of four hemolysin genes (*hla, hlb, hlgC*, and *hld*) were retrieved from the GenBank database. Primer 5.0 software was used to design the primers (Table 1) and these primers were synthesized by Sangon Biotech Co., Ltd. (Shanghai, China).

**Table 1:** **Primers used in this study**.

**Primers**	**Sequence(5′'–3′')**	**Purpose**
F-*hla*	ATGGTGAATCAAAATTGGGG	qRT-PCR
R-*hla*	GTTGTTTGGATGCTTTTC	
F-*hlb*	GCCAAAGCCGAATCTAAG	
R-*hlb*	CGAGTACAGGTGTTTGGT	
F-*hlgC*	CTCTTGCCAATCCGTTATTA	
R-*hlgC*	GCTTTAACATGATTAGTTTT	
F-*hld*	GAGTTGTTTAATTTTAAG	
R-*hld*	TTTTAGTGAATTTGT	
F-*16s*	TGAGATGTTGGGTTAAGTCCCGCA	qRT-PCR for internal control
R-*16s*	CGGTTTCGCTGCCCTTTGTATTGT	
F*-mecA*	AAAATCGATGGTAAAGGTTGGC	Detecting the gene *mecA* by PCR
R*-mecA*	AGTTCTGCAGTACCGGATTTG	
F*-tst*	ATGTCTACAAACGATAAT	Detecting the gene *tst* by PCR
R*-tst*	TTAATTAATTTCTGCTTC	

SPSS 17.0 was used for data analysis. Measurement data is presented as $\bar{x}$ ±*s*. *T*-test for two independent samples was used to compare the relative expression of each of the four hemolysin genes. Statistical significance was defined as *p* < 0.05.

### Detection of α-hemolysin (hla) expression by western blot

The concentration of the SIHP and the reference strain *S. aureus* ATCC25923 were adjusted to 5.0 McFarland using a turbidity meter. Total protein was then extracted from each sample, adjusted to the same concentration and underwent electrophoresis on an SDS-PAGE gel. The proteins were then transferred to a nitrocellulose membrane and the membrane was blocked with 5% skim milk for 1 h. Goat anti-staphylococcal α-hemolysin polyclonal antibodies (Abcam) were added at a final concentration of 2 μg/mL. The reaction mixture was then incubated at 4°C overnight. After the membrane was washed, 1:1000 diluted HRP-labeled rabbit anti-goat IgG antibodies were added and incubated at 37°C for 2 h. Finally, chemiluminescent substrates were added for color development. The expression of α-hemolysin was finally observed under the imager.

### Antimicrobial susceptibility testing of SIHP

The microtiter broth dilution method was used to perform antimicrobial susceptibility screening. The procedure was done according the operational manual of the Phoenix-100 automated microbiology system. The results from this screening were interpreted using the M100-S24 criteria introduced by the Clinical and Laboratory Standards Institute (CLSI; Clinical and Laboratory Standards Institute, 2014). Methicillin-resistant *S. aureus* (MRSA) status was determined using the MIC (minimum inhibitory concentration) of two antibiotics. For the SIHP strains ≥4 μg/mL was the MIC for oxallicin, and ≥8 μg/mL was the MIC for cefoxitin.

### Detection of *mecA* and *tst* genes

All 60 SIHP strains were tested for the drug-resistance gene *mecA* and virulence gene *tst*. Boiling extraction of genomic DNA was conducted following cellular lysis using lysostaphin. The *mecA* and *tst* genes were then amplified from the genomic DNA using the DreamGreen Taq kit. PCR conditions were as follows: pre-denaturation at 94°C for 3 min, denaturation at 94°C for 30 s, annealing at 50°C for 30 s, extension at 72°C for 1 min. PCR products were sequenced and analyzed by BLAST to validate to be the expected products. The sequences of drug-resistance gene *mecA* and virulence gene *tst* of *S. aureus* were retrieved from the GenBank database. Primer 5.0 software was used to design the primers (Table 1) and these primers were synthesized by Sangon Biotech Co., Ltd. (Shanghai, China).

### Detection of the survival of *S. aureus* SIHP strains in macrophages

To compare the survival of *S. aureus* SIHP strains in macrophages with that of *tst* negative *S. aureus* SCHP strains, the human monocyte cell line THP-1 was maintained in RPMI-1640 containing 10% (v/v) FBS at 37°C in an atmosphere containing 5% (v/v) CO_2_. For macrophage infection, THP-1 cells were seeded at 5×10^5^ cells per well in 24-well tissue-culture dishes and induced to differentiate by 10^−7^ M phorbol 12-myristate 13-acetate (PMA) for 48 h. Approximately 3×10^8^ colony-forming units (CFU) of logarithmic phase (OD_600_ 0.5–0.6) bacteria were pelleted by centrifugation, washed twice with PBS, and resuspended in 1 ml of RPMI-1640. Then bacteria were added to the cell monolayer at a MOI of 20:1, and centrifuged for 5 min at 1000 rpm. Infected cells were incubated for 20 min at 37°C, then were washed three times with pre-warmed PBS (pH 7.4) and incubated for an additional 1 h in medium containing 100μg/ml gentamicin to kill extracellular bacteria. Cells were washed and lysed to quantify the intracellular bacterial as the time zero sample (*T*_0_). Additional cells were collected after 12 or 24 h of incubation in the presence of fresh supplemented tissue-culture medium containing 12 μg/ml gentamicin. The growth of bacteria in THP-1 cells was determined by dividing the number of intracellular bacteria at 12 h or 24 h by the number at time 0 (*T*_12_/*T*_0_ or *T*_24_/*T*_0_). The experiment was repeated independently three times.

### Detection of cytokine secretion of macrophages infected by *S. aureus* SIHP strains

The culture supernatants of THP-1 macrophages infected for 12 h by bacteria were collected. The cytokine levels were measured by FACSCalibur flow cytometry (BD) using a CBA Human Thl/Th2 Cytokine Kit II (BD) according to the manufacture's instruction.

### Multilocus sequence typing and analysis

MLST was performed as previously described, including internal fragments of the following seven housekeeping genes: *arcC* (carbamate kinase), *aroE* (shikimate dehydrogenase), *glpF* (glycerol kinase), *gmk*(guanylate kinase), *pta* (phosphate acetyltransferase), *tpi* (triosephosphate isomerase), and *yqiL* (acetyl coenzyme A acetyltransferase) (Heym et al., 2002). PCR amplification of these seven housekeeping genes was done with the primers which sequences were given at the MLST website (http://pubmlst.org/saureus/info/primers.shtml). Amplicons were sequenced by Sangon Biotech Co., Ltd. (Shanghai, China). MLST alleles and sequence types (ST) were determined for each strain with the *S. aureus* MLST website scheme (Profile/ST query) (http://pubmlst.org/perl/bigsdb/bigsdb.pl?db=pubmlst_saureus_seqdef).

## Results

### Hemolytic phenotype of SIHP strains on sheep agar blood plates

**Figure 1:**
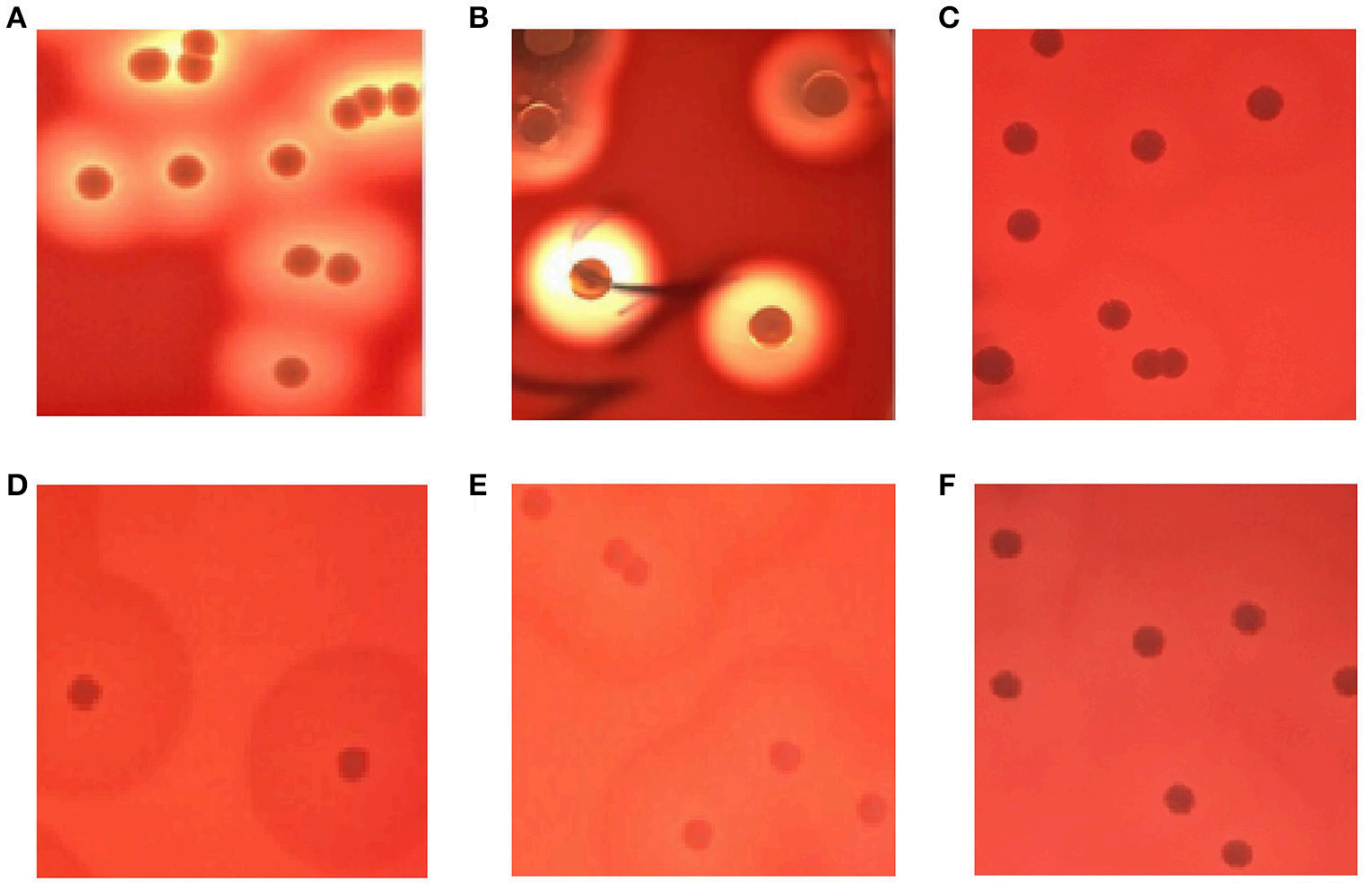
**Hemolytic phenotype comparative analyses of ***S. aureus*** in blood agar plates. (A)** control strain *S. aureus* ATCC25923; **(B)** SCHP; **(C)** SIHP(growing in the microaerophilic condition); **(D)** SIHP(growing in the aerobic condition); **(E)** SIHP(growing in the anaerobic condition); **(F)** SIHP after 10 passages.

As shown in Figures 1A,B, the complete hemolytic ring (β-hemolytic phenotype) was observed in control *S. aureus* strains after culturing strains on blood agar plates for 24 h. However, different hemolytic phenotypes (called incomplete hemolytic phenotype here) were displayed in SIHP strains. Moreover, the incomplete hemolytic phenotype could be observed in the SIHP strains which grown in different environmental conditions including microaerophilic, aerobic and anaerobic conditions respectively (Figures 1C–E). In addition, after 10 serial passages, this incomplete hemolytic phenotype was still maintained (Figure 1F).

### Relative mRNA expression levels of four hemolysin genes

**Figure 2:**
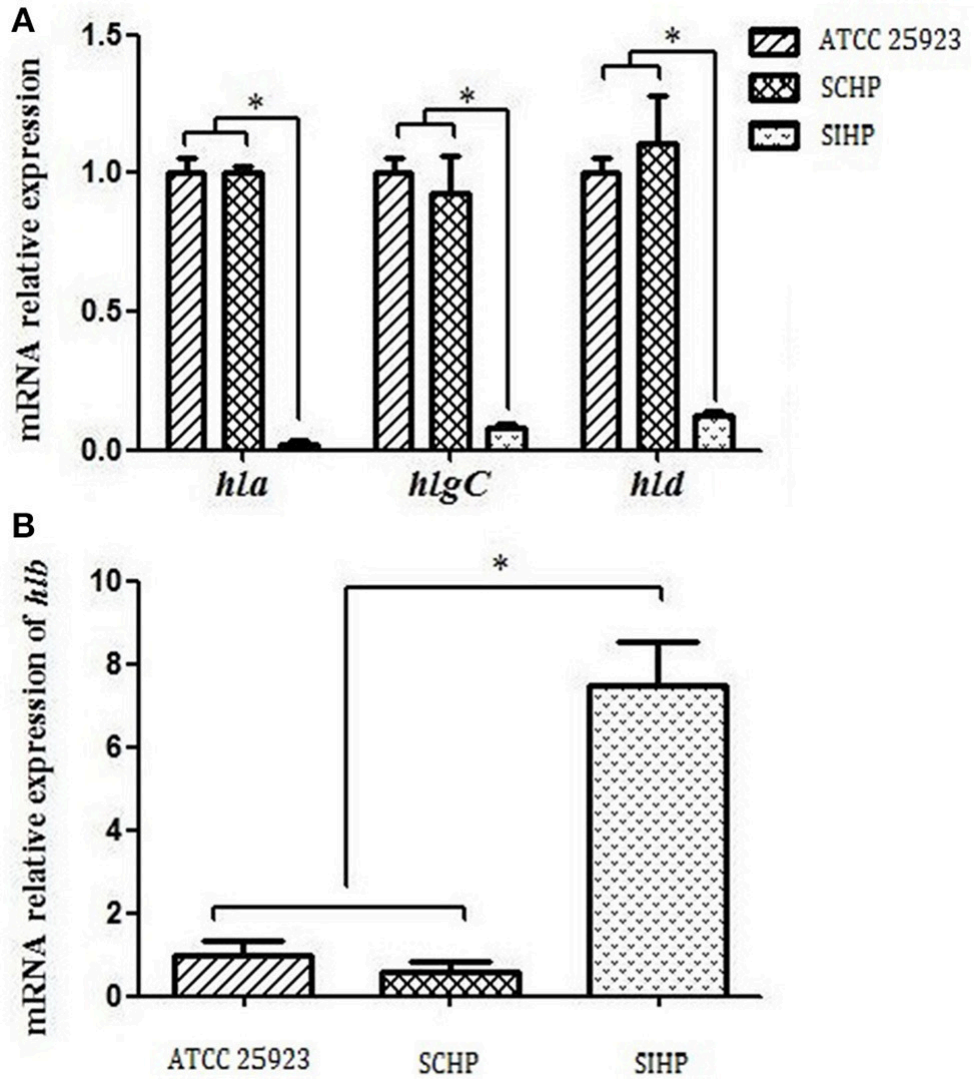
**Comparison of the transcriptional expression of four hemolytic genes in ***S. aureus***. (A)** Expression of *hla, hlgC*, and *hld* in *S. aureus* ATCC25923, SCHP, and SIHP. **(B)** Expression of *hlb* in *S. aureus* ATCC25923, SCHP, and SIHP. ^*^P < 0.05.

As shown in Figure 2, the expression levels of the four hemolysin genes *hla, hlb, hlgC*, and *hld* in SCHP strains exhibited no statistically significant differences (*p* > 0.05) compared to that of control strains ATCC25923. However, the expression levels of *hla, hlgC*, and *hld* in SIHP strains were significantly suppressed by 50-, 16.7-, and 8.3-folds respectively (*p* < 0.05) compared with that of control strains ATCC25923 and SCHP strains, while the expression of *hlb* in SIHP strains was significantly increased 7.7-folds compared to the controls (*p* < 0.05).

### Detection of α-hemolysin by western blot

**Figure 3:**
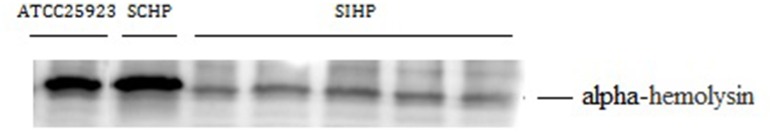
**Western blot of α -hemolysin in ***S. aureus*****. ATCC29213: control *S. aureus* with the complete hemolytic phenotype; SCHP: *S*. *aureus* with the complete hemolytic phenotype isolated from clinical samples in the same period; SIHP: *S*. *aureus* with an incomplete hemolytic phenotype.

The α-hemolysin encoded by gene *hla* is a pore-forming toxin secreted by *S. aureus* and its molecular weight is 33 kDa (Andrey et al., 2015; Al Laham et al., 2015). Western blot analysis showed that the expression of α-hemolysin in SIHP strains was 40-folds lower than that of the SCHP and control strains, which is consistent with the qRT-PCR data (Figure 3).

### Drug resistance of SIHP

Drug susceptibility tested using microtiter broth dilutions demonstrated that the MIC values for oxacillin and cefoxitin were all >2 and >8 μg/mL respectively in 60 SIHP stains. According to the CLSI M100-S24 guidelines, all 60 SIHP strains are classified as MRSA strains. PCR also detected the *mecA* gene in all 60 strains.

### Testing for the *tst* gene

The *tst* gene is an important virulence factor in *S. aureus*. PCR showed that all 50 of the SIHP strains carried *tst* genes.

### The survival ability of *S. aureus* SIHP strains in macrophages is greater than that of *tst* negative *S. aureus*

**Figure 4:**
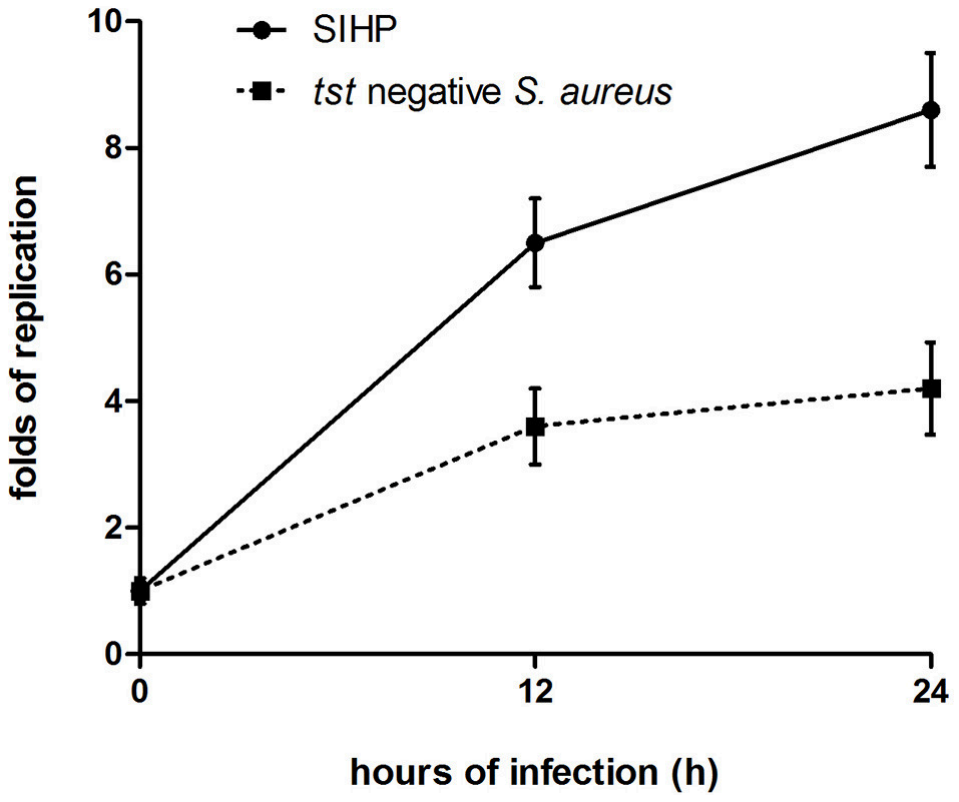
**Survival of SIHP strains and ***tst*** negative ***S. aureus*** in THP-1 derived macrophages**. After the macrophages were infected by bacteria for 12 and 24 h, the macrophages were lysed and the intracellular surviving bacteria were plated on LB agar. The replication folds of intracellular surviving bacteria were calculated according to the numbers of colony on the above LB agar plate.

We compared the intracellular survival abilities of SIHP strains and *tst* negative SCHP strain in macrophages. By counting the number of bacteria recovered from the plates, we found that intracellular survival ability of SIHP strain was much higher than that of *tst* negative *S. aureus* strain after infected THP-1 derived macrophages 12 or 24 h (Figure 4). Preliminarily, we speculated that SIHP strains possess a potential high virulence, and it was suggested that SIHP should be paid more attention in hospital.

### The secretion differences of cytokines and chemokines of macrophages infected by *S. aureus* SIHP strains and *tst* negative *S. aureus*

**Figure 5:**
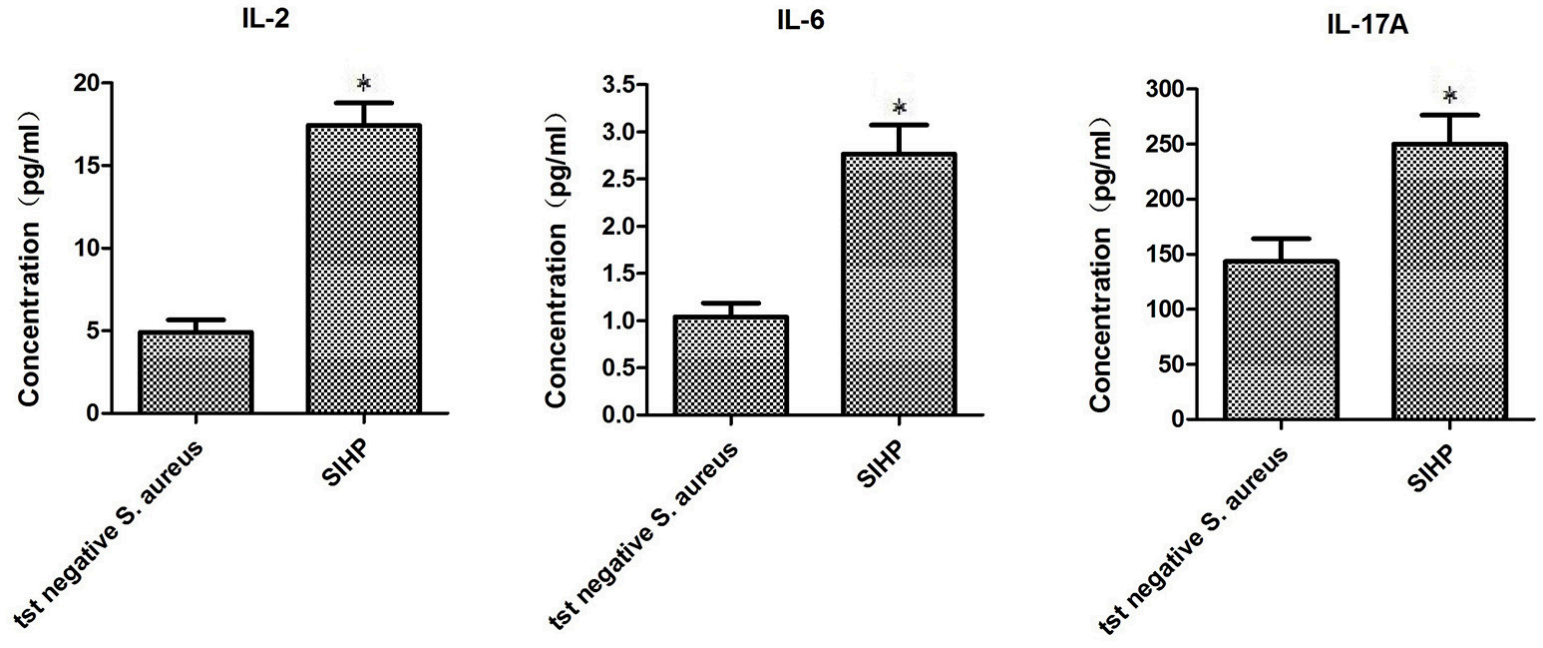
**Detection of cytokines and chemokines secreted by macrophages infected with ***S. aureus*****. ^*^P < 0.05.

To compare the secretion differences of cytokines and chemokines in THP-1 derived macrophages infected by SIHP strain and *tst* negative *S. aureus* strain, we detected pro-inflammatory cytokines by flow cytometry in the culture supernatant of macrophages infected by SIHP strains and *tst* negative *S. aureus* strain. The results showed significantly higher induction of IL-2, IL-6, and IL-17A in SIHP strains, but not by *tst* negative *S. aureus*, suggesting that SIHP strains induce cytokine/chemokine responses in the macrophages (Figure 5). The other cytokines IL-4, IL-10, INF-γ, and TNF secreted by macrophages were no obvious differences between SIHP strains and *tst* negative *S. aureus* affected groups.

### Multilocus sequence typing

We determined the multilocus genotype of 60 SIHP isolates collected from patients admitted in our hospital between 2013 and 2014. The seven-locus scheme recommended in the *S. aureus* MLST database was applied. MLST analysis showed that all of SIHP strains were classified into ST5 clone.

## Discussion

Hemolysin is one of the most important virulence factors for *S. aureus* (Wiseman, 1975). The combined effects of each of the four kinds of hemolysin result in the destruction of the red cell membrane which leads to the formation of the full transparent hemolytic ring on blood agar plates. In recent years, SIHP were found in the clinical samples of the second affiliated hospital of Soochow University. In this study, we sought to identify these strains and study their characteristics in microbiology. Initially, we used different commercial or self-prepared sheep blood agar plates to isolate, incubate and serially passage the strains. We showed that there are some strains that exhibit and maintain an incomplete hemolytic phenotype even after prolonged sub-culture. Thus, the possibility that this phenotype is induced by non-bacterial factors can be excluded. Our studies suggest SIHP could be a subset of unique *S. aureus* strains.

Previous studies suggested that rabbit red blood cells are highly sensitive to α-hemolysin (Hildebrand et al., 1991). Berube et al. reported that the effect of α-hemolysin on red blood cell lysis is concentration-dependent (Berube and Bubeck Wardenburg, 2013). γ-hemolysin can damage the red blood cells of human and animals (Kaneko and Kamio, 2004). While δ-hemolysin damages red blood cells, only at high concentrations, it forms a trans-membrane pore which lyses the cell membrane (Verdon et al., 2009). In this study, we showed that the expression of four hemolysins of SIHP strains and SCHP strains has significantly different transcriptional expression profiles. The expression of α-, γ-, δ-hemolysin in the SIHP strains, which could damage the red cells directly, is much lower than that of the SCHP strains, while β-hemolysin have far higher expression levels compared to the SCHP strains. In addition, the protein expression level of α-hemolysin was further validated by western blot. It was reported that β-hemolysin predominantly increases the sensitivity of red blood cells to other toxins instead of lysing the cells directly, unless the cells are cultured in lower temperatures (Vandenesch et al., 2012). In this study, the high expression of β-hemolysin was detected in SIHP strains, but the complete hemolytic ring was never observed in the SIHP strains even when incubated at 4°C. These results suggest that the observed incomplete phenotype of SIHP strains is most likely the effects of all four hemolysins which are expressed in a different way compared to the SCHP strains. Previous studies have shown, that during chronic pathogenesis, the combined effect of selective pressures resulting from antibiotics including ciprofloxacin and trimethoprim as well as the host immune response can lead to the increased expression of α-hemolysin, which is very important for the colonization of *S. aureus* in mucosa especially in respiratory tract infections (Goerke et al., 2006; Huseby et al., 2010). In this study, all of the patients infected with SIHP received long-term treatment of broad-spectrum antibiotics, whether this selective pressure induced over-expression of drug-resistant genes and interfered with the expression of the hemolysin genes remains a possibility that needs further examination.

To explore the microbiological characteristics of SIHP, we conducted antimicrobial susceptibility tests and chose the virulence gene *tst* which are associated with *S. aureus* pathogenicity. We found that all 60 SIHP strains could be classified as MRSA strains and carried the *mecA* gene. The results of antimicrobial susceptibility tests showed that even after 10 passages, of these 60 SIHP strains, the MIC-values of linezolid and teicoplanin of only three SIHP strains have changed little. However, these changes of MIC values did not influence the assessment of the results of antimicrobial susceptibility. The virulence gene *tst* encodes for toxic shock syndrome toxin (TSST-1) (Kreiswirth, 1989). As a super antigen, TSST-1 enhances the shock and immune suppression responses induced by endotoxins (Kulhankova et al., 2014). PCR testing found that all 60 SIHP strains possessed the *tst* gene suggesting increased virulence. Recently, many reports showed that *S. aureus* could survive in the human monocyte-derived macrophages and these *S. aureus* strains are more virulent (Kubica et al., 2008; Tranchemontagne et al., 2015; Münzenmayer et al., 2016; Nandi and Bishayi, 2016). Therefore, we compared the intracellular survival abilities of SIHP strains and SCHP strains which is *tst* negative *S. aureus* in macrophages, and found that intracellular survival ability of SIHP strain was much higher than that of *tst* negative *S. aureus* strain. In addition, patient data analysis showed that the effect of anti-infective therapy in most of the patients infected with SIHP strains was worse than that of the similar patients infected with SCHP strains (data not shown). Our study provides preliminary experimental evidence for the speculation that SIHP strains possess a potential high virulence.

During the infection process of invasive *S. aureus*, pathogen-associated-molecules initiate the innate immune system leading to activation and recruitment of neutrophils and macrophages and the production of pro-inflammatory cytokines, most notably TNF-α, IL-1β, and IL-6 (Bekeredjian-Ding et al., 2015; Giai et al., 2016; Zhao et al., 2016). It was reported that the secretion of inflammatory cytokines IL-6 increased with increasing concentrations of *S. aureus* (Chen et al., 2016). In addition, superantigenic *S. aureus* are particularly efficient in stimulating IL-17 production (Islander et al., 2010). In this study, SIHP strains showed significantly higher induction of IL-2, IL-6 and IL-17A of macrophages compared to *tst* negative SCHP strains, which suggest that SIHP strains could induce more secretion of cytokines against the killing effect of macrophages. In addition, MLST analysis showed that all of SIHP strains were classified into ST5 clone. Moreover, these SIHP strains were grouped together in the *spa* type of t2460 (data not shown).

Taken together, SIHP may be a new subset of MRSA with potential high virulence, and more attention should be paid in controlling and treatment of these strains in hospitals.

## Author contributions

Conceived and designed the experiments: HaZ, HD, HuZ; Performed the experiments: HaZ, YZ, HG, PX, MW, AL, MM, XX, YD; Analyzed the data: HaZ, HD; Wrote the manuscript: HaZ, HD, YZ.

### Conflict of interest statement

The authors declare that the research was conducted in the absence of any commercial or financial relationships that could be construed as a potential conflict of interest.

## References

[B1] Al Laham N. Mediavilla J. R. Chen L. Abdelateef N. Elamreen F. A. Ginocchio C. C.. (2015). MRSA clonal complex 22 strains harboring toxic shock syndrome toxin (TSST-1) are endemic in the primary hospital in gaza, palestine. PLoS ONE 10:e0120008. 10.1371/journal.pone.0120008 25781188 PMC4364023

[B2] Andrey D. O. Jousselin A. Villanueva M. Renzoni A. Monod A. Barras C.. (2015). Impact of the regulators SigB, Rot, SarA and sarS on the toxic shock Tst promoter and TSST-1 expression in *Staphylococcus aureus*. PLoS ONE 10:e0135579. 10.1371/journal.pone.0135579 26275216 PMC4537247

[B3] Bekeredjian-Ding I. Stein C. Uebele J. (2015). The innate immune response against *Staphylococcus aureus*. Curr. Top. Microbiol. Immunol. [Epub ahead of print]. 10.1007/82_2015_5004. 26667045

[B4] Berube B. J. Bubeck Wardenburg J. (2013). *Staphylococcus aureus* α-toxin: nearly a century of intrigue. Toxins (Basel). 5, 1140–1166. 10.3390/toxins5061140 23888516 PMC3717774

[B5] Changchien C. H. Chen S. W. Chen Y. Y. Chu C. (2016). Antibiotic susceptibility and genomic variations in *Staphylococcus aureus* associated with Skin and Soft Tissue Infection (SSTI) disease groups. BMC Infect Dis. 16:276. 10.1186/s12879-016-1630-z 27287530 PMC4902997

[B6] Chen Q. Hou T. Wu X. Luo F. Xie Z. Xu J. (2016). Knockdown of TNFR1 suppresses expression of TLR2 in the cellular response to *Staphylococcus aureus* infection. Inflammation 39, 798–806. 10.1007/s10753-016-0308-4 26846887

[B7] Clinical and Laboratory Standards Institute (2014). M100-S24 Performance Standards for Antimicrobial Susceptibility Testing; Twenty-Fourth Informational Supplement[S]. Wayne, PA: CLSI.

[B8] den Reijer P. M. Haisma E. M. Lemmens-den Toom N. A. Willemse J. Koning R. A. Demmers J. A.. (2016). Detection of alpha-toxin and other virulence factors in biofilms of *Staphylococcus aureus* on polystyrene and a human epidermal model. PLoS ONE 11:e0145722. 10.1371/journal.pone.0145722 26741798 PMC4704740

[B9] Evangelista Sde S. de Oliveira A. C. (2015). Community-acquired methicillin-resistant *Staphylococcus aureus*: a global problem. Rev. Bras. Enferm. 68, 128–135, 136–143. 10.1590/0034-7167.2015680119p 25946506

[B10] Giai C. Gonzalez C. D. Sabbione F. Garofalo A. Ojeda D. Sordelli D. O.. (2016). *Staphylococcus aureus* induces shedding of IL-1RII in monocytes and neutrophils. J. Innate Immun. 8, 284–298. 10.1159/000443663 26967533 PMC6738831

[B11] Goerke C. Köller J. Wolz C. (2006). Ciprofloxacin and trimethoprim cause phage induction and virulence modulation in *Staphylococcus aureus*. Antimicrob Agents Chemother. 50, 171–177. 10.1128/AAC.50.1.171-177.2006 16377683 PMC1346766

[B12] Heym B. Le Moal M. Armand-Lefevre L. Nicolas-Chanoine M. H. (2002). Multilocus sequence typing (MLST) shows that the ‘Iberian’ clone of methicillin-resistant *Staphylococcus aureus* has spread to France and acquired reduced susceptibility to teicoplanin. J. Antimicrob. Chemother. 50, 323–329. 10.1093/jac/dkf132 12205056

[B13] Hildebrand A. Pohl M. Bhakdi S. (1991). *Staphylococcus aureus* alpha-toxin. Dual mechanism of binding to target cells. J. Biol. Chem. 266, 17195–17200. 1894613

[B14] Huseby M. J. Kruse A. C. Digre J. Kohler P. L. Vocke J. A. Mann E. E.. (2010). Beta toxin catalyzes formation of nucleoprotein matrix in *staphylococcal* biofilms. Proc. Natl. Acad. Sci. U.S.A. 107, 14407–14412. 10.1073/pnas.0911032107 20660751 PMC2922554

[B15] Islander U. Andersson A. Lindberg E. Adlerberth I. Wold A. E. Rudin A. (2010). Superantigenic *Staphylococcus aureus* stimulates production of interleukin-17 from memory but not naive Tcells. Infect. Immun. 78, 381–386. 10.1128/IAI.00724-09 19822653 PMC2798201

[B16] Kaneko J. Kamio Y. (2004). Bacterial two-component and hetero-heptameric pore-forming cytolytic toxins: structures, pore-forming mechanism, and organization of the genes. Biosci. Biotechnol. Biochem. 68, 981–1003. 10.1271/bbb.68.981 15170101

[B17] Kreiswirth B. N. (1989). Genetics and expression of toxic shock syndrome toxin 1: overview. Rev. Infect. Dis. 11(Suppl. 1), S97–S100. 2648542 10.1093/clinids/11.supplement_1.s97

[B18] Kubica M. Guzik K. Koziel J. Zarebski M. Richter W. Gajkowska B.. (2008). A potential new pathway for *Staphylococcus aureus* dissemination: the silent survival of *S. aureus* phagocytosed by human monocyte-derived macrophages. PLoS ONE 3:e1409. 10.1371/journal.pone.0001409 18183290 PMC2169301

[B19] Kulhankova K. King J. Salgado-Pabón W. (2014). *Staphylococcal* toxic shock syndrome: superantigen-mediated enhancement of endotoxin shock and adaptive immune suppression. Immunol Res. 59, 182–187. 10.1007/s12026-014-8538-8 24816557

[B20] Löffler B. Hussain M. Grundmeier M. Brück M. Holzinger D. Varga G.. (2010). *Staphylococcus aureus* panton-valentine leukocidin is a very potent cytotoxic factor for human neutrophils. PLoS Pathog. 6:e1000715. 10.1371/journal.ppat.1000715 20072612 PMC2798753

[B21] Lowy F. D. (1998). *Staphylococcus aureus* infections. N. Engl. J. Med. 339, 520–532. 10.1056/NEJM199808203390806 9709046

[B22] Münzenmayer L. Geiger T. Daiber E. Schulte B. Autenrieth S. E. Fraunholz M.. (2016). Influence of Sae and Agr regulated factors on the escape of *Staphylococcus aureus* from humanmacrophages. Cell Microbiol. 18, 1172–1183. 10.1111/cmi.12577 26895738

[B23] Nandi A. Bishayi B. (2016). Intracellularly survived *Staphylococcus aureus* after phagocytosis are more virulent in inducing cytotoxicity in fresh murine peritoneal macrophages utilizing TLR-2 as a possible target. Microb. Pathog. 97, 131–147. 10.1016/j.micpath.2016.06.007 27270212

[B24] Otto M. (2014). *Staphylococcus aureus* toxins. Curr. Opin. Microbiol. 17, 32–37. 10.1016/j.mib.2013.11.004 24581690 PMC3942668

[B25] Qin N. Tan X. Jiao Y. Liu L. Zhao W. Yang S.. (2014). RNA-Seq-based transcriptome analysis of methicillin-resistant *Staphylococcus aureus* biofilm inhibition byursolic acid and resveratrol. Sci. Rep. 4:5467. 10.1038/srep05467 24970710 PMC4073122

[B26] Tranchemontagne Z. R. Camire R. B. O'Donnell V. J. Baugh J. Burkholder K. M. (2015). *Staphylococcus aureus* strain USA300 perturbs acquisition of lysosomal enzymes and requires phagosomal acidification for survival inside macrophages. Infect. Immun. 84, 241–253. 10.1128/IAI.00704-15 26502911 PMC4694005

[B27] Vandenesch F. Lina G. Henry T. (2012). *Staphylococcus aureus* hemolysins, bi-component leukocidins, and cytolytic peptides: a redundant arsenal of membrane-damaging virulence factors. Front. Cell Infect. Microbiol. 2:12. 10.3389/fcimb.2012.00012 22919604 PMC3417661

[B28] Verdon J. Girardin N. Lacombe C. Berjeaud J. M. Héchard Y. (2009). delta-hemolysin, an update on a membrane-interacting peptide. Peptides 30, 817–823. 10.1016/j.peptides.2008.12.017 19150639

[B29] Voss A. Doebbeling B. N. (1995). The worldwide prevalence of methicillin-resistant *Staphylococcus aureus*. Int. J. Antimicrob. Agents 5, 101–106. 10.1016/0924-8579(94)00036-T 18611655

[B30] Wiseman G. M. (1975). The hemolysins of *Staphylococcus aureus*. Bacteriol. Rev. 39, 317–344. 1108866 10.1128/br.39.4.317-344.1975 PMC408339

[B31] Zhao G. Wu H. Jiang K. Rui G. Zhu Z. Qiu C.. (2016). IFN-τ inhibits *S. aureus*-induced inflammation by suppressing the activation of NF-κB and MAPKs in RAW 264.7 cells and mice with pneumonia. Int. Immunopharmacol. 35, 332–340. 10.1016/j.intimp.2016.02.016 27025553

